# Coding Alone? AI-Assisted Software Work and the Decoupling of Productivity from Public Knowledge-Infrastructure Participation

**DOI:** 10.3390/jintelligence14050089

**Published:** 2026-05-20

**Authors:** Tianhe Jiang

**Affiliations:** Institute of Population Research, Nanjing University of Posts and Telecommunications, Nanjing 210023, China; tianhej@njupt.edu.cn

**Keywords:** generative AI, AI-assisted software development, distributed cognition, collective intelligence, knowledge infrastructure, digital trace data

## Abstract

Complex knowledge work depends on individual output and on public exchanges that document problems, evaluate contributions, route expertise, and preserve reusable knowledge. Software work makes this infrastructure unusually visible through GitHub issues, reviews, comments, mentions, and cross-project ties. As generative AI coding tools become private, on-demand sources of task support, it is unclear whether productive output remains tightly coupled with participation in this GitHub-visible public knowledge infrastructure. This study examines that question using a balanced panel of approximately 38,000 freelance developers on GitHub observed quarterly from 2019 to 2025 (approximately 1,080,000 person-quarter observations), estimating within-person changes in the association between a Productivity Index and a Social Connectivity Index. Two-way fixed effects models estimate a substantively large weakening after mid-2022 (−0.138 SD, about 44 percent of the pre-AI slope), and the pattern remains stable across alternative operationalizations, model specifications, and sample definitions. A survey-linked subsample (*n* = 237) provides individual-level triangulation: the weakening aligns with developers’ self-reported AI adoption dates, and heavier AI users exhibit larger decoupling. Decomposition by exchange function is selective: public exchanges with more direct private AI support pathways (information seeking, troubleshooting, preliminary evaluation) weaken more than exchanges anchored in contextual judgment and new-tie formation. This study documents a large-scale behavioral decoupling between productive output and visible GitHub-based public knowledge-infrastructure participation in a real-world problem-solving setting. The pattern is consistent with cognitive offloading as one micro-level pathway, while direct process evidence is left to future work.

## 1. Introduction

Generative AI has become a routine tool for externally supported problem-solving in knowledge work. Published evidence is accumulating across settings: field experiments with software developers find higher completed-task output when developers receive access to AI coding assistants (Cui et al., 2026); Copilot telemetry studies report productivity-related benefits (Ziegler et al., 2024); professional writers complete writing tasks substantially faster with large language models (Noy & Zhang, 2023); and customer support agents improve productivity when an AI assistant is available (Brynjolfsson et al., 2025). These gains show that AI can improve observable task performance. For intelligence research, they raise a second question: how does AI change the external supports through which complex problem-solving is organized? Work on distributed cognition and collective intelligence points in the same direction: effective problem-solving is rarely solitary. It unfolds across people, artifacts, records, and organized practices (Hutchins, 1995,Woolley et al., 2010).

Software work makes that distributed character unusually visible. Public repositories carry code, issue threads, pull-request reviews, comments, mentions, cross-references, and traces of collaboration across projects. These records form a public-facing layer of the software knowledge infrastructure: they document problems, evaluate contributions, coordinate decisions, route attention to relevant expertise, and leave solutions in place for later reuse. GitHub’s Octoverse reports document rapid growth in platform activity and generative AI-related development (GitHub, 2024). Against that backdrop, the present study asks whether productive output and visible public engagement on GitHub continue to move together: has individual output become less tightly connected to participation in the GitHub-visible public infrastructure that has historically supported shared problem-solving?

Two empirical streams motivate this question. One establishes that AI tools can lift individual productivity (Brynjolfsson et al., 2025; Cui et al., 2026; Noy & Zhang, 2023; Ziegler et al., 2024). The other documents a decline in public knowledge exchange after major AI releases: Stack Overflow question-asking fell sharply after ChatGPT (del Rio-Chanona et al., 2024), and related work documents reduced participation in online developer knowledge communities (Burtch et al., 2024). Related published evidence from creative problem-solving shows that human-guided AI search can substitute for some functions traditionally supplied by human crowds while producing a different performance profile (Boussioux et al., 2024). The open empirical question is whether the relationship between output and public exchange is itself shifting as AI tools diffuse across a working population.

The link between these two streams has largely been treated as a background condition rather than an outcome of interest: individual studies document that AI raises productivity, or that AI reduces public knowledge exchange, but rarely ask whether the within-person coupling between the two is itself shifting. We take that coupling as the object of study. Using roughly 38,000 freelance developers observed quarterly on GitHub from 2019 to 2025, we estimate within-person changes in the association between a Productivity Index (a within-person composite of commits, merged pull requests, code volume, and distinct repositories) and a Social Connectivity Index (an analogous composite of outgoing @-mentions, reviews given, and issue comments on others’ repositories). The productivity–infrastructure link weakens sharply after mid-2022; the timing coincides with major AI tool releases, and in a survey-linked subsample of 237 developers, it tracks each person’s self-reported adoption quarter rather than a shared calendar cutoff. Cognitive offloading offers one micro-level account of how developers allocate effort across supports: once AI becomes a private, on-demand source for retrieval, diagnosis, and preliminary evaluation, some demands that previously moved through public peer exchange may be handled through private AI assistance (Risko & Gilbert, 2016). The empirical object of the study, however, is the behavioral coupling itself rather than the cognitive process behind it.

The paper contributes on three fronts. Empirically, it documents a substantively large and robust weakening in the within-person association between a composite Productivity Index and a Social Connectivity Index after 2022-Q2, with the exact effect size and robustness distribution reported in the Results Section. Methodologically, a three-layer architecture triangulates the pattern—large-scale panel estimates (Layer 1), individual adoption evidence (Layer 2), and decomposition by interaction type and organizational context (Layer 3)—with the inferential reach of each layer stated explicitly. Theoretically, this paper links AI productivity research with distributed-cognition, knowledge-infrastructure, and collective intelligence work by documenting a pattern in which AI-era task performance is less tightly coupled with the public exchanges that maintain reusable knowledge, peer evaluation, and exposure to new perspectives.

This focus is theoretically motivated. Freelance developers serve here as a critical case, not as a proxy for all knowledge workers. They are an early and voluntary adoption setting: developers were among the earliest occupational groups exposed to AI coding assistants at scale, with GitHub reporting rapid growth in AI-related development activity (GitHub, 2024), a diffusion pattern confirmed in recent work (Daniotti et al., 2026). Their GitHub activity is also less shaped by formal review rules, team assignments, or managerial oversight than that of organizationally embedded developers. If the diffusion of AI assistance is accompanied by a looser link between output and public infrastructure participation, this is the population in which the pattern should be most visible. How far it extends to organizational settings or other knowledge-work domains is an empirical question for later work.

The remainder of this paper is organized as follows. Section 2 develops the theoretical framework linking distributed cognition, knowledge infrastructure, collective intelligence, and AI-assisted private problem-solving. Section 3 details the research design, including the population identification pipeline, variable construction, primary econometric specification, event study design, and survey component. Section 4 presents results across the three analytical layers. Section 5 discusses competing interpretations, limitations, and implications.

## 2. Theoretical Framework and Literature Review

### 2.1. Complex Problem-Solving as Distributed and Infrastructured

Distributed-cognition research has long argued that complex problem-solving unfolds across people, artifacts, representations, and organized practices (Hutchins, 1995). Cognitive-artifact and extended-cognition traditions show how external resources make information visible, manipulable, and routinely available for thought (Clark & Chalmers, 1998; Kirsh & Maglio, 1994). Transactive memory adds the social complement: groups develop shared systems for knowing who knows what, so people can encode, retrieve, and coordinate knowledge through one another (Wegner, 1987). Collective intelligence research adds the group-level version of the same point: problem-solving capacity depends on interaction patterns, social sensitivity, and communication structure as well as on members’ individual abilities (Engel et al., 2014; Woolley et al., 2010). These lines of work together locate performance in a cognitive ecology rather than in isolated individual ability.

Knowledge infrastructures are what stabilize that ecology. Infrastructure studies describe them as relational systems embedded in practice and made visible mainly when they change or break down (Star, 1999; Star & Ruhleder, 1996). Knowledge-infrastructure and human-infrastructure research extend the frame to the networks of people, artifacts, institutions, standards, maintenance work, and coordination practices through which knowledge is generated, shared, and reused (Edwards et al., 2013; Lee et al., 2006). Recent work in entrepreneurship extends this view, treating digital infrastructure as an external enabler of cognition and action in innovation processes (Saeedikiya et al., 2024).

The theoretical concern here is the routing of cognitive work in a real problem-solving environment. Generative AI can change where developers seek explanations, diagnoses, examples, and preliminary judgments. If some of those functions move from public exchange to private AI assistance, then the ecology that supports problem-solving changes even when observed output rises. Public knowledge infrastructures carry part of that ecology in visible social form: they help workers define problems, evaluate solutions, and encounter perspectives they would otherwise miss.

### 2.2. Public Knowledge Infrastructure in Software Work

In software work, GitHub provides a public-facing, platform-mediated layer of knowledge infrastructure. Issues document bugs, feature requests, and unresolved design questions. Pull-request reviews evaluate contributions and preserve judgments about code quality. Comments coordinate interpretation across contributors, and mentions route attention to relevant expertise. Cross-project ties and references make dependencies, reusable solutions, and reputational signals visible across project boundaries. These traces are not the whole knowledge infrastructure of software work: developers also communicate through private repositories, Slack, Discord, email, organizational trackers, meetings, documentation, and code itself. They are, however, one unusually observable public layer through which software knowledge becomes visible, linkable, and reusable.

GitHub research supports this infrastructural interpretation. Work on social coding shows that public activity streams and shared project artifacts support collaboration, learning, reputation, and social inference in open software communities (Dabbish et al., 2012). Pull-request evaluation depends on both technical quality and social signals, making review a socio-technical process of evaluation and governance rather than a purely individual coding act (Tsay et al., 2014). Work on socio-technical congruence links coordination gaps to development productivity and software failures, and project diversity shapes software work outcomes (Cataldo & Herbsleb, 2013; Vasilescu et al., 2015). The consistent finding is that public social embeddedness and productive output have historically moved together in software work.

The productive value of these exchanges also follows from social capital theory. Structural holes and weak ties provide access to nonredundant information, expertise, and opportunities (Burt, 1992; Granovetter, 1973), and meta-analytic evidence links network position and social capital to work outcomes including task performance, creativity, career advancement, and firm performance (Fang et al., 2015; Nezami et al., 2025; Stam et al., 2014). In infrastructural terms, social ties help workers access and maintain the public resources through which knowledge work is accomplished.

### 2.3. AI Assistance and Private Problem-Solving Support

The rapid diffusion of generative AI coding assistants since 2022 adds a new private-support channel to this ecology. Published field-experimental evidence among professional developers finds that access to AI coding assistants increases completed-task output, with larger gains for less experienced developers (Cui et al., 2026); published Copilot telemetry evidence likewise reports productivity-related benefits reflected in developer activity data (Ziegler et al., 2024). In professional writing, ChatGPT cut completion time and compressed quality inequality between more and less skilled workers (Noy & Zhang, 2023). In customer support, AI assistance raised average productivity, with gains concentrated among less experienced workers (Brynjolfsson et al., 2025).

The social side of AI diffusion points in a different direction. Stack Overflow question-asking fell sharply after ChatGPT (del Rio-Chanona et al., 2024); related work shows reduced participation in online developer knowledge communities after major AI releases (Burtch et al., 2024). Published experimental evidence in creative problem-solving also shows that human-guided AI can alter the role of human crowds in solution search (Boussioux et al., 2024). These studies together show that AI can move both output and public exchange, yet they leave the within-person relationship between the two largely unexamined.

Cognitive offloading offers one account of how developers allocate effort across supports. People routinely use external resources to lighten the information-processing demands of a task (Risko & Gilbert, 2016), and earlier work on Internet search and smartphones shows how such external knowledge resources can reshape behavior and memory strategy (Barr et al., 2015; Ward, 2013). Once AI coding tools become a private, on-demand source of retrieval, diagnosis, and preliminary evaluation, some demands that previously traveled through public peer exchange may be handled through private AI assistance. Generative AI can also support ideation and creative exploration (Sternberg, 2024; Zhao et al., 2025); the distinction we care about is therefore functional rather than categorical. Some public exchange functions have more direct private AI support pathways; others lean more heavily on social context, unfamiliar perspectives, new-tie formation, and community participation.

### 2.4. From Digital Traces to Public Knowledge-Infrastructure Participation

The empirical object of this study is developers’ visible participation in the public-facing knowledge infrastructure of software work. GitHub records actions rather than mental processes, but those actions are meaningful traces of participation in public systems of problem documentation, evaluation, coordination, and expertise routing. Digital trace data can serve as valid indicators of behavioral constructs when researchers specify how traces are generated, which platform affordances produce them, and where interpretation stops (Salganik, 2018). For the present construct, the validation task is to define which public actions count as visible participation in shared problem documentation, evaluation, coordination, and attention routing; it is not to infer private cognition directly from any single trace.

We treat GitHub indicators as behavioral traces of public knowledge-infrastructure participation. Each trace can serve multiple functions, and no single trace maps one-to-one onto a cognitive process. Table 1 identifies the infrastructure function used in our analysis, the possible cognitive-social role, important alternative functions, and the inferential boundary attached to each indicator.

The inferential force of the design comes from the aggregate pattern rather than from any single trace. A question-bearing comment may express information seeking, coordination, or community participation; a code review may involve routine evaluation, mentoring, signaling, or governance. This study therefore estimates changes in the relationship between productive output and a composite index of public infrastructure participation, then triangulates the pattern through event study timing, individual AI adoption evidence, survey help-seeking responses, and decomposition by public exchange function. The mapping is therefore a bridge from public action to publicly enacted cognitive-social work; it does not run from trace data to private mental states. Cognitive interpretations are explanatory claims about why the pattern may arise, separate from the measured construct itself.

For the decomposition analysis, these trace categories are implemented with transparent rule-based tags rather than intent classification. Question-bearing comments are issue comments with question or help-seeking markers; troubleshooting mentions are @-mentions in issue or pull-request threads containing bug, error, failure, or fix language; cross-domain contributions are contributions outside a developer’s established repository or language profile; and first-contact engagements are first-observed outgoing interactions with a previously unseen account in the public work graph. These rules group public exchange functions for analysis; they do not claim to recover the private motive behind any single action or to provide validated intent-classifier precision and recall.

### 2.5. Hypotheses

The theoretical logic yields two testable predictions. The first concerns the overall relationship between output and public infrastructure participation. In the pre-AI regime, productive quarters should also be more publicly connected quarters for the same developer: more commits, merged pull requests, and repository activity typically coincide with more reviews, issue comments, mentions, and cross-project interaction. AI assistance introduces a private-support channel that can help sustain output without requiring the same level of visible public exchange.

**H1** **(Productivity–infrastructure decoupling).** *The within-person association between productive output (PI) and public knowledge-infrastructure participation (SCI) weakens after the mass availability of generative AI coding tools.*

The second prediction concerns selectivity. AI tools provide more direct private support for some public exchange functions than for others. Information seeking, troubleshooting, and preliminary evaluation can often be supported through model-generated suggestions, explanations, and candidate fixes. Boundary-crossing, first-contact engagement, and exposure to nonredundant perspectives depend more heavily on social context, public visibility, and relationship formation.

**H2** **(Heterogeneity by public exchange function).** *The weakening is stronger for public exchange functions with more direct private AI support pathways, such as information seeking, troubleshooting, and preliminary evaluation, than for functions more dependent on contextual exposure and new ties.*

H1 and H2 correspond, respectively, to the aggregate decoupling finding and the decomposition analysis reported in Section 4.1 and Section 4.4. The direction of the relationship remains empirical. AI could strengthen the productivity–infrastructure coupling if it frees developers for deeper collaboration, creates new interaction needs around AI-generated artifacts (Ziegler et al., 2024), or enables more ambitious projects requiring greater coordination. The direction, magnitude, and selectivity of the shift are therefore open questions to be answered at the population scale.

The hypotheses treat public GitHub traces as visible participation in the knowledge infrastructure of software work and AI assistance as a private-support channel whose role may expand after tool diffusion. Cognitive offloading is one micro-level pathway within this account: developers may route some immediate information-seeking, diagnostic, or preliminary evaluative demands to AI tools when those tools are cheaper or more convenient than public peer exchange.

The design that follows operationalizes these hypotheses on the population where the test is most informative: freelance developers whose GitHub interactions and AI adoption are both voluntary and publicly observable.

## 3. Research Design and Methods

### 3.1. Population Identification: Freelance Developers on GitHub

As discussed in the Introduction Section, freelance developers offer the cleanest setting for this test: without imposed collaborative structures or employer-directed AI policies, every interaction on GitHub is a behavioral choice. GitHub also records each commit, pull request, review, issue comment, and @-mention with precise timestamps, giving the design the richest behavioral trace data available for any knowledge-work population. Because this study uses public GitHub.com platform records rather than GitHub Enterprise Server or a locally installed software package, no single stable public application version number applies; GitHub Enterprise Server and the GitHub REST API are versioned separately. All GitHub traces refer to public GitHub.com platform records observed for the 2019–2025 study window.

Picking freelance developers out of GitHub’s population of more than 100 million accounts calls for a classification pipeline that balances precision against recall. Freelancer status is rarely labeled at the account level, so the goal is not a perfect occupational label but a transparent, auditable population definition whose reach can be tested against plausible classification thresholds.

Our approach proceeds in four stages. Stage 1 constructs seed labels using high-precision identity signals. Positive labels are assigned to accounts whose company field contains multilingual keywords such as “freelance,” “self-employed,” or “digital nomad,” whose bio contains phrases such as “available for hire” or “contractor,” or whose composite score across these indicators reaches or exceeds 0.5. Negative labels are assigned to accounts with verified membership in known corporate GitHub organizations, corporate company fields, or corporate email domains. Accounts with fewer than fifty total commits between 2019 and 2025 are excluded, as are bot accounts identified through a username-pattern and commit-uniformity heuristic and ambiguous academic accounts flagged by .edu email domains combined with academic bio content and fewer than three non-academic repositories.

Stage 2 engineers twelve behavioral features across three dimensions (temporal, project, and identity) that distinguish freelance from employed work patterns without relying on network variables that would create circularity with the outcome measure.

Table 2 summarizes these twelve features and their anticipated relationships with freelancer status. Temporal features capture the irregular, distributed work rhythms typical of freelance developers who serve multiple clients across timezones. Project features capture the portfolio-style engagement characteristic of freelancing. Identity features capture profile presentation patterns associated with self-marketing. To keep the sampling layer auditable, feature coding is deliberately rule-based: identity composites use field-presence indicators, temporal drift features are computed from changes in the modal commit-hour profile, and project features are derived from repository-tenure and language counts before any SCI variables are constructed.

Stage 3 trains a supervised classifier using XGBoost (v3.1.3), with Optuna (v3.4) used only as an automated hyperparameter search procedure over 200 trials. In non-technical terms, the classifier learns which combinations of work timing, project diversity, and profile presentation best distinguish the high-confidence positive and negative seed labels. The planned performance targets were an area under the receiver operating characteristic curve exceeding 0.85 and an F1 score exceeding 0.75; the realized hold-out results exceed both thresholds.

Stage 4 subjects the classifier to validation checks that combine supervised test-set performance with external reference points: enterprise false-positive verification, freelance platform cross-reference using Upwork and Fiverr profile matching, Stack Overflow developer survey self-reports, and threshold sensitivity in the main specification. Table 3 reports the planned validation criteria alongside the observed diagnostics.

The remaining risk worth naming is misclassification: some independent contractors read as employees, and some employees present as freelancers. The validation diagnostics reduce this concern but do not eliminate it. The threat is largest if errors correlate with post-2022 shifts in social interaction or with time-varying identity presentation. Because the classifier uses observable profile and behavioral histories rather than an exogenous occupational registry, the sample should be read as an analytic freelancer panel rather than as error-free occupational ground truth. The threshold variants (Pr(freelancer)≥0.3, Pr(freelancer)≥0.5, and Pr(freelancer)≥0.7) reduce our dependence on any single cutoff, though they leave systematic classification drift as a residual limitation we flag here rather than dismiss.

### 3.2. Core Variables

#### 3.2.1. Productivity Index

The Productivity Index (PI) is a composite of four components, each log-transformed as ln(x+1) before within-person standardization: commit count, merged pull requests, code volume (insertions plus deletions), and number of distinct repositories contributed to. All within-person standardization uses each developer’s pre-2022-Q2 mean and standard deviation as the reference baseline. The equal-weight average of these standardized components constitutes ${\mathrm{PI}}_{\mathrm{full}}$, the primary productivity measure. A secondary specification, ${\mathrm{PI}}_{\mathrm{merged}}$, retains only merged pull requests, a measure that requires human review and is therefore more resistant to inflation by AI-generated code.

PI captures observable productive output in development work: the tangible artifacts (code, merged contributions, repository activity) that result from a developer’s work, whether that work was supported by public peer exchange, AI assistance, or individual problem-solving.

A natural concern is that AI inflates productivity metrics (more commits, more code volume), mechanically weakening the PI-SCI correlation. This concern is substantively important rather than merely technical: if AI creates a pathway to productive output that does not require visible collaborative interaction, the PI-SCI link may weaken partly because productivity now has a larger non-social component. The change in what PI captures is therefore part of what the paper must document carefully. That said, if $\beta_{2}$ holds when productivity is measured exclusively through merged pull requests, which require another human to review, evaluate, and approve the contribution, the result cannot be attributed to inflated commit counts.

An important temporal distinction bears on this concern. Throughout the bulk of the study period (2022-Q2 through mid-2025), the dominant AI coding tools (GitHub Copilot’s inline autocomplete, ChatGPT, and Claude) operated as assistive technologies: they suggested code completions or answered queries in a conversational interface, but every commit remained a human-initiated action. Agentic tools capable of autonomously creating branches, writing code, committing changes, and opening pull requests, such as GitHub Copilot Coding Agent (GitHub, 2025) and Claude Code (Anthropic, 2025), reached general availability only in mid-to-late 2025, covering at most the final one to two quarters of our observation window. As an additional robustness check, restricting the observation window to end at 2025-Q2, before any agentic AI tool reached general availability, yields substantively identical results ($\beta_{2}=-0.134$, SE =0.010).

#### 3.2.2. Social Connectivity Index

The Social Connectivity Index (SCI) operationalizes visible participation in GitHub’s public-facing knowledge infrastructure (see Section 2.4 for the trace-to-function mapping). The primary measure, ${\mathrm{SCI}}_{\mathrm{outgoing}}$, is a within-person *z*-scored composite of three outgoing interaction types: @-mentions sent to other developers, code reviews given on others’ pull requests, and issue comments posted on repositories owned by others. These components capture public attention routing, distributed evaluation, information exchange, and knowledge coordination across project boundaries. The choice of outgoing interactions as the primary measure follows the theoretical prediction: if AI assistance supplies private support for some problem-solving demands, developers may sustain output with fewer outbound contributions to public peer exchange. A robustness specification, ${\mathrm{SCI}}_{\mathrm{full}}$, adds incoming interactions and network structure variables (unique interaction partners, reciprocal ties).

SCI captures public-facing collaborative behavior on GitHub, not the totality of developers’ professional social interaction or the full knowledge infrastructure of software work. Developers communicate via Slack, Discord, email, video calls, private repositories, organizational trackers, and in-person meetings. A decline in SCI is therefore consistent with reduced public participation, migration to private channels, or both. We address this boundary through a platform-migration diagnostic within the SCI decomposition. That diagnostic uses linkable Stack Overflow activity for the subset with matched accounts; because it is narrower and lacks one-to-one GitHub component counts, it is treated as a directional check rather than as a decomposition component. Table 4 consolidates the variable definitions, specifications, and analytical roles used throughout the paper.

#### 3.2.3. The Activity-Level Confound

More active developers mechanically register higher values on both PI and SCI, generating a spurious positive correlation that must be addressed to avoid confounding genuine behavioral change with compositional shifts in platform activity. Following methodological warnings about ratio-variable pathologies (Kronmal, 1993) and recommendations for organizational research (Certo et al., 2020), we adopt a tiered approach. The primary specification (Tier 1) uses the unscaled dependent variable with $\log({\mathrm{active}\_\mathrm{days}}_{it})$ as an explicit control, thereby absorbing the mechanical correlation between overall platform activity and both PI and SCI. The secondary specification (Tier 2) decomposes the effect into extensive and intensive margins, distinguishing between changes in how many developers interact at all and changes in how much interacting developers interact. If both the Tier 1 and Tier 2 estimates of $\beta_{2}$ point to an intensive-margin effect, the weakening reflects genuine within-person behavioral change rather than compositional shifts in the active developer population.

### 3.3. Primary Econometric Specification

The core analytical strategy can be stated intuitively: we compare each developer with their own past behavior, asking whether the same individual shows a weaker productivity–public infrastructure link after AI tools became available than before. Two-way fixed effects (TWFE) models formalize this within-person comparison while simultaneously controlling for anything that affects all developers in a given quarter (e.g., macroeconomic conditions, platform-wide changes).

The primary specification takes the form of a two-way fixed effects model with an interaction term capturing the change in the PI–SCI relationship after AI tool availability:

(1) $${\mathrm{SCI}}_{\mathrm{outgoing},it}=\alpha_{i}+\lambda_{t}+\beta_{1}\cdot {\mathrm{PI}}_{it}+\beta_{2}\cdot \left({\mathrm{PI}}_{it}\times {\mathrm{Post}}_{t}\right)+\gamma \cdot \log\left({\mathrm{active}\_\mathrm{days}}_{it}\right)+\varepsilon_{it},$$

where $\alpha_{i}$ denotes developer fixed effects absorbing all time-invariant individual heterogeneity, $\lambda_{t}$ denotes quarter fixed effects absorbing common temporal shocks, ${\mathrm{PI}}_{it}$ is the within-person standardized Productivity Index, ${\mathrm{Post}}_{t}$ is a binary indicator equal to one from 2022-Q2 onward, γ controls for the activity-level confound, and $\varepsilon_{it}$ is the idiosyncratic error term clustered at the developer level. All estimation is conducted in R (v4.3) using the fixest package (v0.12).

The coefficient of primary interest is $\beta_{2}$. A negative and substantively meaningful $\beta_{2}$ indicates that the within-person association between productive output and public knowledge-infrastructure participation weakens after AI tool availability. The coefficient $\beta_{1}$ captures the baseline PI–SCI association in the pre-AI period and is expected to be positive, reflecting the documented coupling between productivity and public social embeddedness in software work.

In practical terms, the SESOI of 0.05 SD corresponds to approximately one to two fewer cross-repository interactions per developer per month: roughly the difference between publicly asking one additional question, reviewing one contribution, or routing one issue to a peer, and handling the same problem outside the public infrastructure. Effects smaller than this threshold are unlikely to reflect meaningful changes in public participation at the individual level.

With an anticipated sample of 600,000 to 1,200,000 person-quarter observations, conventional null hypothesis significance testing is uninformative: virtually any non-zero effect will achieve statistical significance. Following recent methodological guidance for large-sample observational research (Lakens, 2017), we anchor inference in a pre-specified smallest effect size of interest (SESOI) of 0.05 standard deviations of within-person SCI variation. Our decision rule classifies outcomes into three categories: if $|\beta_{2}|>\mathrm{SESOI}$, the decoupling hypothesis is supported; if the ninety percent confidence interval falls entirely within the region of practical equivalence as determined by two one-sided tests (TOSTs), the hypothesis is rejected; if neither condition is met, the result is classified as inconclusive. The specification curve analysis further requires that at least eighty percent of analytical variants produce estimates exceeding SESOI for the finding to be considered robust.

### 3.4. Event Study Design

The event study design is analogous to an interrupted time series analysis familiar in psychological research (Shadish et al., 2002): we examine the trajectory of the productivity–infrastructure relationship before and after a discrete event (AI tool release), testing whether any change coincides with the event rather than reflecting a pre-existing trend.

The event study specification provides temporal resolution on when the PI–SCI weakening begins, enabling assessment of whether the onset coincides with AI tool availability rather than occurring gradually or at other calendar dates. The main reported coefficients center on Copilot General Availability (June 2022), with the Claude 3.5 Sonnet release (June 2024) and a 2021-Q2 placebo date retained as auxiliary timing checks.

(2) $${\mathrm{SCI}}_{it}=\alpha_{i}+\lambda_{t}+\beta_{1}\cdot {\mathrm{PI}}_{it}+\sum_{\begin{array}{c} k=-4\\ k\neq -1 \end{array}}^{+6}\delta_{k}\cdot \left({\mathrm{PI}}_{it}\times 1\left[t-t^{*}=k\right]\right)+\gamma \cdot \log\left({\mathrm{active}\_\mathrm{days}}_{it}\right)+\varepsilon_{it},$$

where $t^{*}$ denotes the release quarter and k=−1 is the omitted reference period. The event-time coefficients $\delta_{k}$ capture how the PI–SCI slope differs from the reference quarter at each relative quarter, so the plotted trajectory is a change in coupling rather than a level shift in SCI.

Unless otherwise noted, the primary estimates use the full balanced 2019–2025 panel. The descriptive pre/post table uses the local 2021-Q1–2022-Q1 period as the immediate pre-AI baseline, and the specification curve separately reports the shorter analytic-window variants.

### 3.5. Survey Component: Individual-Level AI Adoption Evidence

The full-sample analysis documents the panel pattern and shows that its timing is aligned with AI tool releases. But the period after mid-2022 bundles AI tool availability with several concurrent changes: COVID recovery, technology sector layoffs, remote work normalization, and GitHub platform changes. The survey therefore serves mainly as triangulation by adding each developer’s personal AI adoption date and usage intensity.

We draw a stratified random sample from the identified panel rather than from social media, developer forums, or any general recruitment channel. Stratification proceeds along four dimensions: pre-AI SCI level, activity level, primary programming language, and GitHub tenure. The questionnaire comprises ten items organized in four blocks, with a target completion time of four minutes. Q2 and Q3 are the two items the entire survey design exists to collect—the former provides the individual adoption date for the staggered event study, and the latter provides the dose variable for dose–response analysis. Q3 records daily AI use intensity in ordered response bands; the reported high–low contrast defines heavy users as those reporting more than one hour per day and light users as those reporting less than thirty minutes per day, with intermediate categories retained in the adoption-timing analysis but not used for that binary contrast.

**Figure 1:**
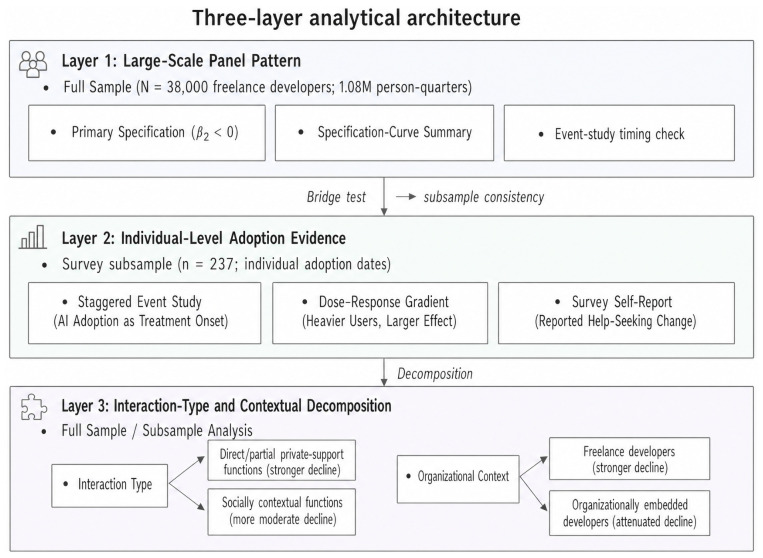
Three-layer analytical architecture. Layer 1 establishes the large-scale panel pattern through the primary specification, specification curve, and event study on the full panel (approximately 38,000 developers; 1.08M person-quarters). A bridge test assesses consistency between the full sample and the survey subsample. Layer 2 triangulates the interpretation with individual-level adoption evidence through staggered event study, dose–response gradient, and survey self-report (n=237). Layer 3 decomposes the decline by interaction type and organizational context.

The three-layer analysis architecture is illustrated in Figure 1. Layer 1 (large-scale panel pattern) establishes the primary finding through the full-sample regression, specification curve, and event study. A bridge test assesses external consistency between the full sample and the survey subsample. Layer 2 (individual-level adoption evidence) replaces the common ${\mathrm{Post}}_{t}$ with individual ${\mathrm{Post}}_{\mathrm{individual},it}$:

(3) $${\mathrm{SCI}}_{it}=\alpha_{i}+\lambda_{t}+\beta_{1}\cdot {\mathrm{PI}}_{it}+\beta_{2}\cdot \left({\mathrm{PI}}_{it}\times {\mathrm{Post}}_{\mathrm{individual},it}\right)+\gamma X_{it}+\varepsilon_{it},$$

and introduces a dose–response test interacting $\beta_{2}$ with AI usage intensity:

(4) $$\begin{array}{c} {\mathrm{SCI}}_{it}=\alpha_{i}+\lambda_{t}+\beta_{1}\cdot {\mathrm{PI}}_{it}+\beta_{2}\cdot \left({\mathrm{PI}}_{it}\times {\mathrm{Post}}_{\mathrm{individual},it}\right)\\ \;\;\;\;\;\;\;\;\;\;\;+\beta_{3}\cdot \left({\mathrm{PI}}_{it}\times {\mathrm{Post}}_{\mathrm{individual},it}\times {\mathrm{Intensity}}_{i}\right)+\gamma X_{it}+\varepsilon_{it}. \end{array}$$

Layer 3 (interaction type and contextual decomposition) disaggregates the overall decline by public exchange function, distinguishing functions with more direct private AI support pathways from functions more dependent on social context and new ties, and by organizational context, comparing freelance developers with organizationally embedded developers.

### 3.6. Specification Curve and Robustness Design

Researcher degrees of freedom (choices about variable operationalization, sample definition, and model specification) can influence results in any single analysis. To address this concern, we conduct a specification curve analysis (Simonsohn et al., 2020), a technique closely related to multiverse analysis in psychology (Steegen et al., 2016): rather than reporting a single “preferred” specification, we systematically vary all defensible analytical choices and report the distribution of results across the entire decision space.

Table 5 summarizes the 288 unique specifications generated by crossing seven analytical dimensions. The specification curve serves a dual purpose: assessing the robustness of the primary finding to researcher degrees of freedom and mapping the regions of the analytical space where the finding is strongest or weakest.

### 3.7. Placebo Tests and Falsification Criteria

Five pre-specified falsification criteria guard against overclaiming. First, if $\beta_{2}$ falls inside the region of practical equivalence across eighty percent or more of specification curve variants, the phenomenon is absent. Second, if $\beta_{2}$ is significant only without the activity-level control but falls inside the ROPE in Tier 1 and Tier 2 specifications, the finding reflects activity composition shift rather than a genuine productivity–infrastructure decoupling. Third, if the event study shows the decline beginning well before any AI tool release, the timing is inconsistent with AI diffusion as a precipitating context. Fourth, if the staggered adoption test in Layer 2 shows no association between the PI–SCI decline and individual adoption dates, the individual-level AI link is unsupported. Fifth, if the staggered event study reveals that the PI–SCI decline begins before the self-reported adoption date, reverse causality cannot be excluded.

## 4. Results

### 4.1. The Phenomenon: Weakening of the Productivity–Infrastructure Correlation

**Figure 2:**
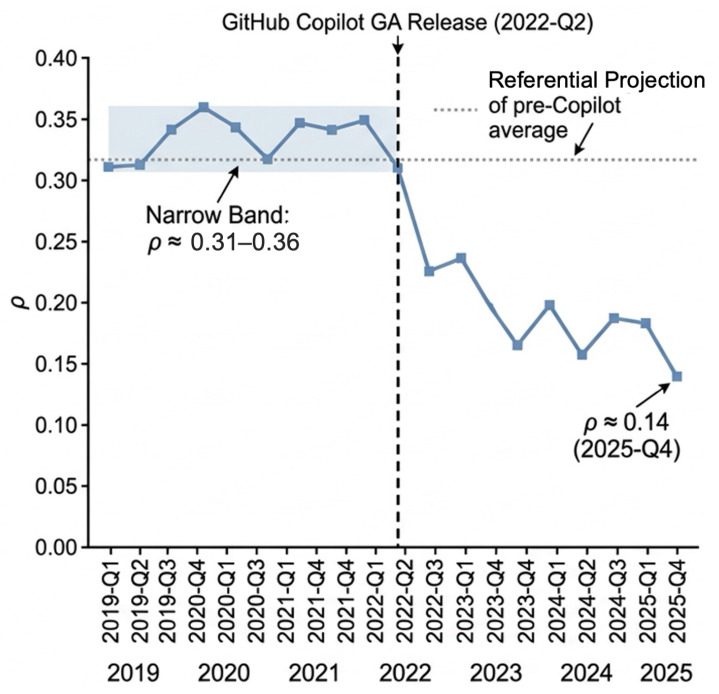
Quarterly Pearson correlation between the Productivity Index and Social Connectivity Index, 2019-Q1 through 2025-Q4. SCI operationalizes visible participation in GitHub’s public-facing knowledge infrastructure. The vertical dashed line marks GitHub Copilot General Availability (2022-Q2); the shaded band indicates the pre-AI baseline range; the horizontal dotted line projects the pre-Copilot average for visual reference.

The quarterly Pearson correlation between ${\mathrm{PI}}_{\mathrm{full}}$ and ${\mathrm{SCI}}_{\mathrm{outgoing}}$, computed across all freelance developers with at least ten active days in a given quarter, is stable between 2019 and early 2022, fluctuating in a narrow band between 0.31 and 0.36. Beginning in 2022-Q2, the correlation drops steeply, reaching 0.24 by 2024-Q4 and 0.14 by 2025-Q4—a decline of approximately 0.20 correlation units, or more than half of the pre-AI baseline. The trajectory is not monotonically declining; minor upward fluctuations appear in 2023-Q1 and 2024-Q1, perhaps reflecting seasonal patterns. But the overall trend is unmistakable, and the inflection point is visually sharp (see Figure 2).

The descriptive statistics in Table 6 reveal a telling asymmetry. Productive output increases across both periods (commits rise from a quarterly mean of 89.3 to 118.7, and merged pull requests from 12.1 to 14.9), while all three components of SCI decline: reviews given fall from 6.3 to 4.7 per quarter, issue comments on others’ repositories from 4.8 to 3.2, and @-mentions sent from 8.1 to 5.9. The number of unique interaction partners per quarter drops from 7.4 to 5.6, a decline of roughly twenty-four percent.

The regression in Table 7 turns this descriptive pattern into a within-person estimate: the productivity–infrastructure coupling weakens by $\beta_{2}=-0.138$ standard deviations (SE =0.009, p<0.001) after Copilot’s general availability, reducing the pre-AI slope by about forty-four percent, well above the pre-specified SESOI of 0.05 SD. Using the SESOI calibration above, this corresponds roughly to three to five fewer cross-repository public interactions per developer per month. Column 1 reports the baseline specification without the interaction term, confirming the expected positive PI–SCI association: a one-standard-deviation rise in within-person productivity is associated with a 0.247 SD rise in outgoing public infrastructure participation ($\beta_{1}$), conditional on individual and quarter fixed effects plus the activity-level control. Column 2 adds the interaction with ${\mathrm{Post}}_{t}$ and returns the $\beta_{2}$ headline figure above.

Column 3 replaces ${\mathrm{PI}}_{\mathrm{full}}$ with ${\mathrm{PI}}_{\mathrm{merged}}$, shrinking the interaction to $\beta_{2}=-0.091$ (SE =0.011). The coefficient still exceeds SESOI, but roughly one-third of the original magnitude is absorbed. We read the attenuation as substantively informative: merged pull requests require human review, so the effect cannot be reduced to inflated commit counts; yet the gap between the two columns also shows that what the Productivity Index captures is itself shifting across the AI transition. Column 4 swaps ${\mathrm{SCI}}_{\mathrm{full}}$ in for ${\mathrm{SCI}}_{\mathrm{outgoing}}$ and yields $\beta_{2}=-0.112$ (SE =0.010). Column 5 applies the Tier 2 activity decomposition and attributes about sixty-eight percent of the overall effect to the intensive margin.

The specification curve analysis, spanning all 288 variants defined in Table 5, shows that 91.3% of specifications produce $|\beta_{2}|>\mathrm{SESOI}$, comfortably exceeding the eighty percent threshold. No specification produces a positive $\beta_{2}$.

**Figure 3:**
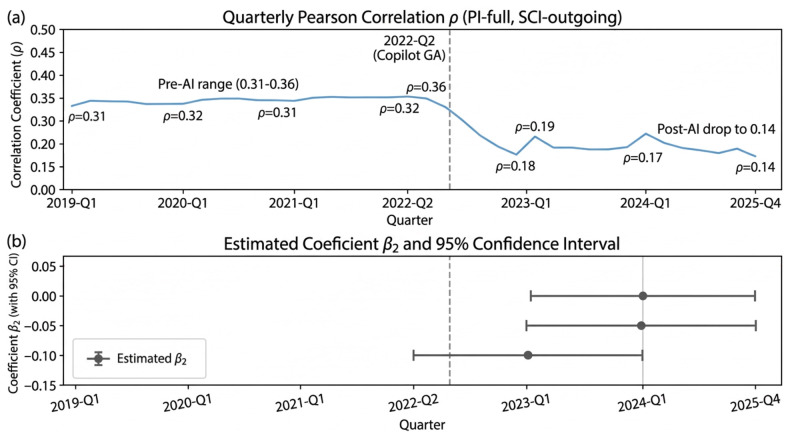
Sub-window robustness checks. Panel (**a**) reproduces the quarterly PI–SCI correlation trajectory for orientation. Panel (**b**) reports within-person interaction estimates ($\beta_{2}$) from three post-AI sub-windows: 2022-Q2–2023-Q1, 2023-Q1–2024-Q1, and 2024-Q1–2025-Q4. Points show estimates; horizontal bars show 95% confidence intervals.

As a separate temporal robustness check, Figure 3 reports the descriptive correlation trajectory and post-AI sub-window estimates.

### 4.2. Timing: Event Study Evidence

**Figure 4:**
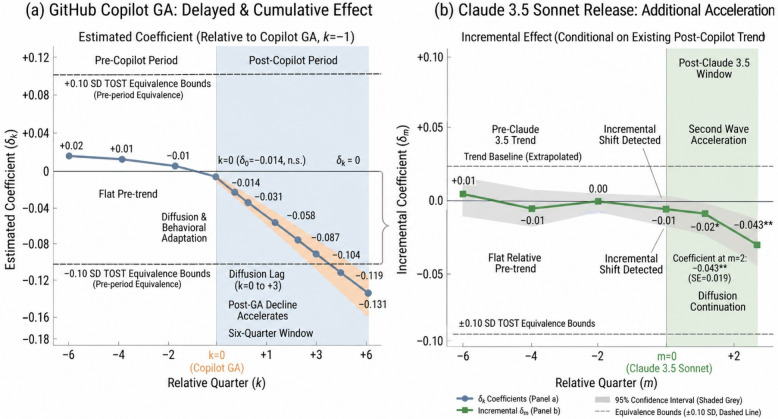
Event study estimates of the PI–SCI coupling by relative quarter. The omitted reference period is k=−1. Pre-release coefficients remain near zero; post-release coefficients become increasingly negative over subsequent quarters. Asterisks, where shown in the figure and accompanying event-study table, indicate significance levels (* p<0.05; ** p<0.01), and n.s. indicates not significant. The orange shaded band in Panel (**a**) highlights the post-GA diffusion/adaptation path, and the green shaded band in Panel (**b**) marks the post-Claude 3.5 window.

The event study (Figure 4, Table 8) provides temporal resolution on the onset of the PI–SCI weakening. The Copilot-centered pre-treatment coefficients in relative quarters k=−4 through k=−1 are collectively indistinguishable from zero under the pre-trend TOST stability bound of ±0.10 SD; the headline SESOI for post-release effect sizes remains 0.05 SD. The post-treatment trajectory shows a gradual but cumulative departure that deepens through subsequent quarters. This timing is consistent with AI diffusion, although it does not by itself rule out all contemporaneous changes in GitHub use or developer labor markets.

The onset at k=0 is muted, with the effect becoming statistically significant only at k=+2 and substantively large by k=+4. This delayed onset is consistent with a diffusion interpretation: Copilot became generally available in June 2022, but widespread adoption among freelance developers took several months. Auxiliary timing checks point in the same direction: the second wave centered on Claude 3.5 Sonnet (2024-Q2) suggests additional acceleration, while the 2021-Q2 placebo estimate falls comfortably inside the ROPE.

### 4.3. Individual-Level Evidence: Survey Subsample

In the survey subsample, individual AI adoption timing is aligned with the population pattern at a larger magnitude: $\beta_{2}=-0.168$ (SE =0.038) on $\mathrm{PI}\times {\mathrm{Post}}_{\mathrm{individual}}$. Of 2743 invitations sent, 237 developers completed the survey and provided valid GitHub usernames that matched the panel for a response rate of 8.6 percent, within the anticipated range. For the full invited set, we observe behavioral strata, activity measures, language, tenure, and invitation metadata; psychosocial self-reports are available only for the 237 respondents. The responder–nonresponder balance table therefore compares observable GitHub behavior rather than unobserved psychosocial traits and shows a modest tilt toward more socially active developers, the direction anticipated under the conservative-bias argument.

A bridge test confirms that the subsample behaves like the panel it came from: the IPW-weighted primary specification on the 237 respondents yields $\beta_{2}=-0.131$ (SE =0.041), statistically indistinguishable from the full-sample estimate.

**Figure 5:**
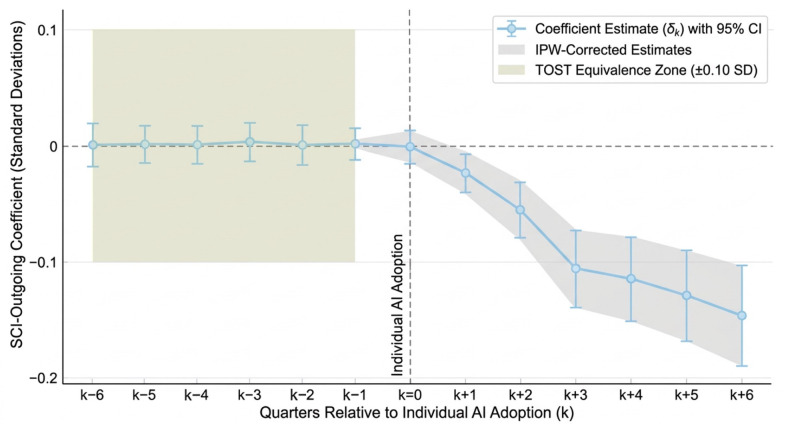
Staggered event study estimates using individual AI adoption dates (survey subsample, n=237). Pre-adoption coefficients cluster near zero; post-adoption coefficients turn negative and strengthen over subsequent quarters. The gray ribbon denotes IPW-corrected estimates; the pale-yellow pre-adoption shaded zone marks the ±0.10 SD TOST equivalence region.

Self-reported AI adoption dates among the 237 respondents span 2021-Q4 (early Copilot beta testers) through 2025-Q3, with the median at 2023-Q1. The staggered event study (Figure 5, Table 9) adds a triangulation check: pre-treatment coefficients are statistically equivalent to zero under TOST, and post-treatment coefficients turn negative and deepen quarter by quarter.

That the staggered estimate of −0.168 sits above the full-sample calendar-time estimate of −0.138 is the direction the conservative-bias argument would predict. Since adoption timing is self-reported rather than randomized, we treat this coefficient as triangulating rather than causal. The dose–response test adds a second triangulation check: the triple interaction is $\beta_{3}=-0.057$ (SE =0.018, p<0.01), meaning that respondents reporting more than an hour of daily AI use show a PI–SCI weakening roughly forty percent larger than those reporting under thirty minutes.

Help-seeking self-reports complement these patterns. When asked whether their frequency of “asking other developers for help” had changed since adopting AI coding tools, 58.2 percent of respondents chose “somewhat less” or “much less.” The open-text responses are consistent with that pattern. A plurality described AI tools as a “first resort” that removed the immediate need to ask peers; one respondent wrote, “I used to ping colleagues on Slack for quick questions about unfamiliar APIs—now I just ask Claude.” A smaller group expressed ambivalence: “I worry that I am losing touch with the community. I used to learn so much from reading other people’s issues and pull requests. Now I just generate code and move on”.

### 4.4. Decomposition: Which Public Exchange Functions Decline?

Exchanges with more direct private AI support pathways weaken more in standardized beta terms than exchanges anchored in context, novelty, or new ties: across the five interaction types we resolve, the mean absolute $\beta_{2}$ is 0.091 for the direct/partial private-support group and 0.045 for the socially contextual group. Decomposing SCI-outgoing by component type (Table 10) makes that asymmetry visible and traces which public functions of software work shifted most sharply with AI diffusion.

The decomposition is consistent with H2 while keeping the construct-validity caveats of Section 2.4 in view. Interactions with more direct private AI support pathways (public knowledge seeking via question-bearing comments, −43.0%; attention routing for diagnosis via troubleshooting mentions, −37.2%; and public evaluation via code reviews, −25.8%) show larger absolute $\beta_{2}$ values than boundary-crossing (cross-domain contributions, −17.5%) and new-tie formation (first-contact engagements, −18.5%). Averaged across the direct and partial private-support rows, $|\beta_{2}|=0.091$; averaged across the socially contextual rows, $|\beta_{2}|=0.045$. The pattern is consistent with selective substitution into private channels for some functions, while public exchange anchored in context and new ties continues. Code review is worth flagging here: it is multi-functional in practice, carrying mentoring, coordination, signaling, relationship maintenance, and contextual judgment alongside routine evaluation, which is part of why we treat it as a “partial” private-support category. The smaller but non-zero decline among the socially contextual rows could reflect a spillover, where reduced engagement for information-seeking indirectly cuts exposure to exploratory opportunities, or a broader shift in the ecology of platform-based work. Distinguishing these would require off-platform interaction data and direct cognitive process measures that our design does not capture.

A platform-migration diagnostic adds nuance. Among the SCI-outgoing components, code reviews are the most plausible candidate for IDE-based absorption. Issue comments on others’ repositories and cross-project @-mentions have no direct IDE substitute, but GitHub Discussions could absorb some question-bearing issue activity. The decline’s extension to reviews and @-mentions therefore argues against a simple IDE-migration story, while leaving on-platform reorganization as a residual boundary condition.

## 5. Discussion and Conclusions

### 5.1. Interpretation of Findings

The paper’s strongest claim is behavioral. A freelance developer who ships more commits, merged pull requests, and repository activity after mid-2022 is no longer the same developer who consistently asks more questions, routes more @-mentions, or weighs in on more of others’ pull requests. Output became less coupled to visible GitHub-based public participation, and the pattern matters because that public layer is one of the ways software work, as complex knowledge work, distributes problem articulation, evaluation, attention routing, reusable knowledge, and exposure to unfamiliar perspectives across people and records.

An AI-linked reading is suggested from several directions at once: the timing of the onset, each developer’s self-reported adoption quarter, the dose–response on usage intensity, and the functional selectivity in the decomposition. Together, these patterns are consistent with an AI-linked interpretation while retaining the limits of an observational design. Platform redesign, private-channel migration, labor-market turbulence, and productivity measurement drift remain live alternatives and deserve to be treated that way, as we do in the Competing Interpretations Section.

The decomposition adds shape to this picture. The functions with the most direct private AI support pathways (public knowledge seeking, troubleshooting attention-routing, and preliminary evaluation) weaken most, while functions that hinge on context, novelty, or new ties weaken more modestly. Cognitive offloading is a natural micro-level pathway for this selectivity, but our evidence describes the large-scale behavioral shift rather than the cognitive process itself.

### 5.2. Limitations and Alternative Accounts

Four design gaps shape how these findings should be read. The first is the public-layer boundary: SCI measures visible GitHub-specific public participation, one observable slice of a larger professional social world that also runs through private repositories, Slack, Discord, email, organizational trackers, and adjacent GitHub channels such as Discussions. This boundary is substantive rather than merely technical: public knowledge infrastructure depends on records that are visible, searchable, linkable, and reusable, so a shift away from GitHub-visible exchange can matter even if some collaboration continues elsewhere. The cross-platform check with Stack Overflow and the within-SCI decomposition argue against a simple platform-migration story, but private and adjacent channels lie outside our window, and some of the decline may reflect where developers’ work becomes visible rather than how much collaboration takes place. Rule-based decomposition tags add a narrower measurement caveat: if issue-comment wording changed after AI diffusion, question-bearing labels may undercount some post-AI help-seeking even when the broader SCI components are unaffected. The second is productivity measurement drift: AI changes what the Productivity Index captures. Pre-AI commits were predominantly human-authored, whereas post-AI commits increasingly incorporate AI-generated code that may differ in quality, originality, and depth of understanding. The ${\mathrm{PI}}_{\mathrm{merged}}$ specification, which requires human review for a contribution to count, attenuates but preserves the main effect, and we read that attenuation as substantively informative. Acknowledging this measurement instability is part of documenting the phenomenon honestly rather than treating it as a nuisance to be explained away. The third is adoption endogeneity: individual AI adoption timing is a behavioral choice rather than a randomization, and unobserved individual characteristics can move both variables together even when the staggered pre-trends look flat. The fourth concerns the cognitive mechanism itself: the measured construct is public knowledge-infrastructure participation, and cognitive offloading is an interpretive pathway that sits alongside it. Observing the mechanism directly would need think-aloud protocols, experience sampling, or controlled manipulation of AI versus peer support, all of which we leave for future work.

Beyond these design boundaries, several alternative accounts of the observed pattern deserve direct engagement.

GitHub platform changes are the most serious competitor. Between 2022 and 2025, GitHub deepened Copilot’s IDE integration, expanded GitHub Actions automation, introduced Copilot Chat inside the web interface itself, and continued to route some repository Q&A through GitHub Discussions rather than issue threads. Any of these could trim observable social interaction on the platform without AI fundamentally changing how developers engage with peers. The SCI decomposition makes a pure-visibility reading less sufficient because the decline extends beyond question-bearing issue comments to reviews and cross-project @-mentions. Even so, the entanglement is genuine: Copilot integration and Discussions are simultaneously changes in tool support and platform visibility, and our design cannot fully disentangle “AI changes how developers work” from “platform reorganization changes where developers’ work is visible”.

COVID-19 recovery and remote-work normalization present a temporal coincidence that the full-sample analysis alone cannot settle. The individual adoption evidence blunts the threat of a purely common-time shock, since reported adoption dates span several years. Cohort and selection confounding remain live, however: early adopters may differ in age, labor-market exposure, remote-work routines, or platform habits that were themselves in flux during this period.

Technology sector layoffs in late 2022 and 2023 reached freelance developers indirectly through the contract market. The PI–SCI weakening we document is a within-person change in the association between productivity and public infrastructure participation rather than a level shift in either variable, and the dose–response gradient on individual AI intensity is awkward to explain with layoff dynamics alone. Labor-market turbulence can still correlate with both contracting opportunities and AI uptake, and we treat it as part of the period context rather than an alternative we have dismissed.

The possibility that experienced developers naturally need less peer input is absorbed by developer fixed effects, argued against by the placebo test in the pre-AI window, and sits awkwardly with the abrupt onset of the shift. Survivorship bias in the balanced panel points the opposite way: requiring activity in twenty-eight consecutive quarters selects for an unusually persistent population, and if the most extreme cases of private AI-assisted work lead to full platform disengagement, the balanced panel will understate the population-level effect. The unbalanced-panel variant in the specification curve produces modestly larger estimates, consistent with that reading.

### 5.3. Contributions and Implications

The study’s main contribution is empirical: a large-scale within-person weakening of the relationship between productive output and participation in GitHub’s public layer of software-work knowledge infrastructure. That finding gives distributed-cognition and knowledge-infrastructure research a measurable field setting, one where changes in private tool support can be studied alongside visible shifts in shared records, reviews, comments, mentions, and cross-project ties.

The results also speak to collective intelligence research. Collective intelligence depends on interaction patterns as much as on individual ability (Engel et al., 2014; Woolley et al., 2010). Rather than measuring group intelligence outcomes directly, the study traces change in one public exchange system that supports evaluation, attention routing, and exposure to nonredundant perspectives. The more moderate decline among boundary-crossing and new-tie interactions is reassuring; that they still decline at all leaves open whether collective capacity is being sustained through other channels or gradually reconfigured.

Cognitive offloading appears here as a plausible micro-level pathway, while the measured construct is public knowledge-infrastructure participation. The offloading literature has mostly examined shifts from internal processing to external artifacts such as calculators, search engines, and smartphones (Barr et al., 2015; Risko & Gilbert, 2016; Ward, 2013). AI-assisted software work raises a neighboring possibility: some information-seeking, diagnostic, or preliminary evaluative demands move from public peer exchange to private machine assistance. The selectivity we see in the decomposition is consistent with that pathway, though pinning it down will take direct process evidence.

A longer-term concern attaches to the public knowledge commons. Declines in Stack Overflow question-asking and comparable forums are usually read as evidence that generative AI now retrieves and synthesizes information previously obtained through public Q&A (Burtch et al., 2024; del Rio-Chanona et al., 2024). If fewer developers contribute questions, answers, issue discussions, and review comments, the public knowledge base that supports learning, community onboarding, and future model training can lose freshness and diversity over time. We frame this as an infrastructural risk to monitor over the longer horizon.

At the same time, the observed decoupling should not be read as evidence of a simple collapse in collaboration or as an intrinsically harmful change. Private AI support may reduce repetitive, low-value help-seeking and allow developers to reserve public interaction for questions that require context, judgment, or relationship-specific knowledge. The concern is therefore more precise: whether public, reusable records, weak-tie exposure, peer learning, and onboarding pathways are maintained as some routine support moves into private AI channels.

Organizational settings form the natural boundary case. A supplementary employee comparison yields $\beta_{2}=-0.072$ (SE =0.006): negative and significant, but roughly half the freelancer magnitude. Team assignments, managerial oversight, formal review rules, and shared repositories likely buffer the decoupling. Freelancers are best read as the critical case where individual choice is most visible, providing an upper-bound rather than a population-wide estimate. The generalization is therefore analytic rather than universal: institutional structures appear to moderate, rather than erase, the decoupling. Extension to other knowledge-work settings is also bounded by visibility: writing, customer support, and analyst work generate far less public infrastructure than software work, so the present design speaks most directly to domains with comparable public artifacts (open scientific repositories, public policy drafting platforms, open data analysis communities) and only suggestively to settings where most exchange is private.

Two threads of future work follow naturally. The developmental stakes belong to one of them: public interaction in professional communities has long served as training ground for explanation, evaluation, critique, perspective taking, and institutional navigation, and whether reduced public help-seeking affects those skills will depend on whether comparable practice is maintained through teams, education, mentoring, or other channels. The dose–response result makes this especially relevant for high-intensity AI users, for whom light-touch mentoring, peer-review, or public help-seeking routines may matter most as substitutes for informal community practice. Concretely, this would mean preserving mandatory human code review even when AI can pre-screen contributions, scheduling rotational mentoring or pair-programming for developers reporting heavy daily AI use, and treating sustained public participation (issue triage, review credit, cross-project referrals) as a recognized contribution rather than an unmonitored side activity, so that the developmental functions of public exchange are not silently eroded by individual efficiency gains. The other thread is direct mechanism testing. Think-aloud protocols could show whether developers now consult AI at moments where they previously would have asked peers; experience-sampling designs could record help-seeking channel choice in real time across AI, private search, and human consultation; controlled experiments could vary the availability, cost, or quality of AI versus peer support and watch how the same problem-solving demand gets routed; and organizational field studies could examine whether formal code review, mentorship, or team norms keep public exchange intact once AI tools are widely adopted.

### 5.4. Conclusions

Human intelligence has always had a social and infrastructural dimension. The capacity to think, solve problems, and produce knowledge has long been entangled with the capacity to interact, consult, record, evaluate, and reuse shared knowledge, a connection captured in work on transactive memory, distributed cognition, and collective intelligence (Hutchins, 1995; Wegner, 1987; Woolley et al., 2010). The finding reported here is that, in one GitHub-visible real-world problem-solving setting, the behavioral link between productive output and public knowledge-infrastructure participation is weakening.

The restructuring stops short of collapse. Developers continue to interact, and the interactions most closely tied to contextual exposure and new ties decline more moderately than functions with more direct private AI support pathways. The trajectory has yet to plateau, and the consequences for public knowledge maintenance, collective capacity, peer learning, and professional judgment are still unfolding.

For intelligence research, the phenomenon opens a distinctive set of questions. How is collective intelligence sustained when public knowledge infrastructure is supplemented by private AI assistance? Which public exchange functions can be partly supported privately by AI, and which depend irreducibly on public visibility, reciprocity, and social context? How should education, platforms, and organizations preserve the developmental value of peer exchange when AI makes some forms of help-seeking feel optional? These questions warrant continued attention as AI continues to reshape the external supports on which complex problem-solving depends.

## Figures and Tables

**Table 1:** Behavioral traces of public knowledge-infrastructure participation.

GitHub Behavioral Trace	Knowledge-Infrastructure Function	Possible Cognitive-Social Role	Alternative Functions	Inferential Boundary
Question-bearing issue comments	Public problem articulation and knowledge seeking	Information retrieval, help-seeking	Coordination, community participation, status updating	Captures visible routing of a knowledge gap to peers; the private reason for asking is not observed
Code reviews given on others’ PRs	Public evaluation and quality governance	Evaluative judgment, mentoring	Signaling, relationship maintenance, project governance	Captures participation in distributed evaluation; covers both routine and substantive judgment
Troubleshooting @-mentions	Attention routing to relevant expertise	Diagnostic support, escalation	Accountability, coordination, notification	Captures recruitment of peer attention to a problem; cognitive dependence is not directly inferred
Cross-domain contributions	Boundary-crossing across problem spaces	Exposure to unfamiliar domains	Career building, opportunistic work, portfolio diversification	Captures public movement across domains; indicates potential exposure to unfamiliar problem spaces
First-contact engagements	New-tie formation in the public work graph	Network expansion, nonredundant exposure	Reputation building, client search, community onboarding	Captures creation of a new visible tie; durable relationship quality is not assessed

**Table 2:** Freelancer classification: behavioral features and expected directions.

Dimension	Feature	Description	Expected Direction
Temporal	A1: Work hour entropy	Shannon entropy of commit hour distribution	Higher for freelancers
Temporal	A2: Weekend ratio	Proportion of commits on weekends	Higher for freelancers
Temporal	A3: Timezone drift count	Number of distinct timezone shifts per year	Higher for freelancers
Temporal	A4: Timezone drift magnitude	Average hours shifted per drift event	Higher for freelancers
Temporal	A5: Session regularity CV	Coefficient of variation in inter-session intervals	Higher for freelancers
Project	B1: Median repo tenure	Median months contributing to each repository	Lower for freelancers
Project	B2: Repo tenure CV	Variability in repository engagement duration	Higher for freelancers
Project	B3: Tech stack breadth	Number of distinct primary languages across repos	Higher for freelancers
Project	B4: Burstiness	Goh–Barabási index (Goh & Barabási, 2008) of commit timing	Higher for freelancers
Project	B5: Multi-project day ratio	Proportion of active days with commits to 2+ repos	Higher for freelancers
Identity	D1: Profile completeness	Composite of bio, location, website, company fields	Higher for freelancers
Identity	D2: Has portfolio	Binary indicator for portfolio/personal site link	Higher for freelancers

**Table 3:** Freelancer classifier: validation criteria and observed diagnostics.

Diagnostic	Planned Criterion	Observed Diagnostic
Hold-out supervised performance	AUC > 0.85; F1 > 0.75; precision and recall examined	AUC = 0.893; F1 = 0.791; precision = 0.812; recall = 0.772; accuracy = 0.843 on a stratified 20% hold-out split of seed-labeled accounts
Enterprise false-positive check	False-positive rate below five percent among known corporate accounts	Observed false-positive rate = 3.1% among 18,425 held-out corporate accounts
Upwork/Fiverr cross-reference	Recall above 0.80 among externally matched freelance profiles	Among 1487 GitHub accounts matched to public Upwork or Fiverr profiles, 82.6% were classified as freelancers at Pr(freelancer)≥0.5
Stack Overflow self-report cross-check	Agreement with available self-reported employment status	Among 2341 Stack Overflow accounts linkable to GitHub through user-supplied profile URLs and carrying Developer Survey employment self-reports, classifier–self-report agreement =82.4% (Cohen’s κ=0.68)
Threshold sensitivity	Main result should not depend on one classification cutoff	$\beta_{2}=-0.131$ at Pr(freelancer)≥0.3, −0.138 at Pr(freelancer)≥0.5, and −0.144 at Pr(freelancer)≥0.7; all exceed the SESOI

*Note.* These diagnostics indicate that the classifier met the planned validation thresholds, but classification error remains an explicit limitation. The Stack Overflow cross-check uses only users who voluntarily exposed a GitHub URL or username in their public Stack Overflow profile, so it functions as an external validation subset rather than a population frame. The classifier is therefore treated as a transparent sampling device with threshold sensitivity, not as error-free ground truth. Threshold variants address dependence on a single cutoff; they do not rule out systematic or time-varying misclassification, such as post-2022 changes in how employee, contractor, or freelancer identities are presented on GitHub.

**Table 4:** Variable definitions and analytical specifications.

Variable	Specification	Components	Analytical Role
${\mathrm{PI}}_{\mathrm{full}}$	Primary	Commits, merged PRs, code volume, repos contributed to (within-person *z*-scored, equal weight)	Primary DV interaction term
${\mathrm{PI}}_{\mathrm{merged}}$	Robustness	Merged PRs only (within-person *z*-scored)	AI inflation check
${\mathrm{SCI}}_{\mathrm{outgoing}}$	Primary	@-mentions sent, reviews given, issue comments to others’ repos (within-person *z*-scored)	Primary DV
${\mathrm{SCI}}_{\mathrm{full}}$	Robustness	${\mathrm{SCI}}_{\mathrm{outgoing}}$ + incoming interactions + network structure	Broader construct check
${\mathrm{Post}}_{t}$	Primary	Binary: 1 if ≥ 2022-Q2 (Copilot GA)	Time indicator
${\mathrm{Post}}_{\mathrm{individual},it}$	Survey subsample	Binary: 1 if ≥ developer *i*’s self-reported AI adoption quarter	Staggered adoption indicator
Active days	Control	$\log({\mathrm{active}\_\mathrm{days}}_{it})$	Activity-level confound
${\mathrm{AIExposure}}_{t}$	Continuous variant	Industry-level AI coding tool penetration rate	Gradual treatment

**Table 5:** Specification curve dimensions and analytical variants.

Dimension	Variations	No. of Variants
Productivity measure	${\mathrm{PI}}_{\mathrm{full}}$, ${\mathrm{PI}}_{\mathrm{merged}}$	2
Social connectivity measure	${\mathrm{SCI}}_{\mathrm{outgoing}}$, ${\mathrm{SCI}}_{\mathrm{full}}$	2
Activity confound approach	Tier 1 (explicit control), Tier 2 (margin decomposition)	2
Classifier threshold	Pr(freelancer)≥0.3, Pr(freelancer)≥0.5, Pr(freelancer)≥0.7	3
Time window	Analytic (2021–2025), Full (2019–2025)	2
Post definition	Binary Copilot GA, Binary ChatGPT, Continuous AIExposure	3
Panel structure	Balanced, Full (unbalanced)	2
Total unique specifications		288

**Table 6:** Descriptive statistics: Analytic window, pre- and post-AI periods.

Variable	Pre-AI Mean (2021Q1–2022Q1)	Pre-AI SD	Post-AI Mean (2022Q2–2025Q4)	Post-AI SD
${\mathrm{PI}}_{\mathrm{full}}$ (within-person *z*)	0.000	1.000	0.187	1.084
${\mathrm{PI}}_{\mathrm{merged}}$ (within-person *z*)	0.000	1.000	0.098	1.031
${\mathrm{SCI}}_{\mathrm{outgoing}}$ (within-person *z*)	0.000	1.000	−0.142	0.962
${\mathrm{SCI}}_{\mathrm{full}}$ (within-person *z*)	0.000	1.000	−0.091	0.978
Active days per quarter	28.4	18.7	31.2	19.3
Commits per quarter	89.3	112.6	118.7	141.2
Merged PRs per quarter	12.1	16.8	14.9	19.4
Reviews given per quarter	6.3	9.1	4.7	7.8
Issue comments (others’ repos)	4.8	7.2	3.2	5.9
@-mentions sent per quarter	8.1	11.4	5.9	9.6
Unique interaction partners	7.4	6.8	5.6	5.9
ρ(${\mathrm{PI}}_{\mathrm{full}}$, ${\mathrm{SCI}}_{\mathrm{outgoing}}$)	0.34	—	0.19	—
Observations (person-quarters)	∼190,000		∼570,000	
Unique developers	∼38,000		∼38,000	

**Table 7:** Primary regression results: productivity–infrastructure decoupling.

	(1) Baseline	(2) ${\mathrm{PI}}_{\mathrm{full}}\times \mathrm{Post}$	(3) ${\mathrm{PI}}_{\mathrm{merged}}\times \mathrm{Post}$	(4) ${\mathrm{SCI}}_{\mathrm{full}}$	(5) Tier 2 Intensive
${\mathrm{PI}}_{it}$	0.247 ***	0.312 ***	0.284 ***	0.273 ***	0.298 ***
	(0.007)	(0.009)	(0.010)	(0.008)	(0.010)
${\mathrm{PI}}_{it}\times {\mathrm{Post}}_{t}$	—	−0.138 ***	−0.091 ***	−0.112 ***	−0.094 ***
		(0.009)	(0.011)	(0.010)	(0.012)
$\log({\mathrm{active}\_\mathrm{days}}_{it})$	0.184 ***	0.179 ***	0.181 ***	0.193 ***	—
	(0.005)	(0.005)	(0.005)	(0.005)	
Developer FE	Yes	Yes	Yes	Yes	Yes
Quarter FE	Yes	Yes	Yes	Yes	Yes
Observations	∼1,080,000	∼1,080,000	∼1,080,000	∼1,080,000	∼764,000
$R^{2}$ (within)	0.089	0.094	0.087	0.091	0.082
DV	SCI-out	SCI-out	SCI-out	SCI-full	SCI-out
PI measure	${\mathrm{PI}}_{\mathrm{full}}$	${\mathrm{PI}}_{\mathrm{full}}$	${\mathrm{PI}}_{\mathrm{merged}}$	${\mathrm{PI}}_{\mathrm{full}}$	${\mathrm{PI}}_{\mathrm{full}}$

*Note*. *** *p* < 0.001. Standard errors clustered at the developer level in parentheses. All specifications include developer and quarter fixed effects.

**Table 8:** Event study coefficients around Copilot general availability (2022-Q2).

Relative Quarter	$\delta_{k}$	SE	95% CI	TOST Equivalence
k=−4	0.008	0.013	[−0.017,0.033]	Equivalent
k=−3	−0.011	0.012	[−0.035,0.013]	Equivalent
k=−2	0.018	0.013	[−0.007,0.043]	Equivalent
k=−1	(ref.)	—	—	—
k=0	−0.014	0.012	[−0.037,0.009]	—
k=+1	−0.031 **	0.014	[−0.058,−0.004]	—
k=+2	−0.058 ***	0.016	[−0.089,−0.027]	—
k=+3	−0.087 ***	0.018	[−0.122,−0.052]	—
k=+4	−0.104 ***	0.020	[−0.143,−0.065]	—
k=+5	−0.119 ***	0.022	[−0.162,−0.076]	—
k=+6	−0.131 ***	0.024	[−0.178,−0.084]	—

*Note.* ** p<0.01; *** p<0.001. Pre-trend TOST equivalence bounds set at ±0.10 SD. k=−1 is the reference period.

**Table 9:** Staggered event study: individual adoption dates (survey subsample, n=237).

Relative Quarter	$\delta_{k}$	SE	95% CI	Pre-Trend TOST
k=−4	0.019	0.034	[−0.048,0.086]	Equivalent
k=−3	−0.024	0.031	[−0.085,0.037]	Equivalent
k=−2	0.038	0.033	[−0.027,0.103]	Equivalent
k=−1	(ref.)	—	—	—
k=0	−0.028	0.032	[−0.091,0.035]	—
k=+1	−0.064 ^†^	0.035	[−0.133,0.005]	—
k=+2	−0.103 **	0.038	[−0.178,−0.028]	—
k=+3	−0.139 ***	0.041	[−0.219,−0.059]	—
k=+4	−0.161 ***	0.046	[−0.251,−0.071]	—
k=+5	−0.174 ***	0.051	[−0.274,−0.074]	—
k=+6	−0.183 ***	0.056	[−0.293,−0.073]	—

*Note.* ^†^ p<0.10; ** p<0.01; *** p<0.001. Treatment onset is each developer’s self-reported AI adoption quarter. Pre-trend TOST equivalence bounds set at ±0.10 SD.

**Table 10:** Decomposition of productivity–infrastructure decoupling by public exchange function.

Interaction Type	Public Exchange Function	AI Support Pathway	Pre-AI	Post-AI	Δ%	$\beta_{2}$
Question-bearing comments	Public knowledge seeking	Direct private support	2.14	1.22	−43.0%	−0.098
Troubleshooting @-mentions	Attention routing for diagnosis	Direct private support	3.47	2.18	−37.2%	−0.091
Code reviews given	Public evaluation and quality governance	Partial private support	6.31	4.68	−25.8%	−0.083
Cross-domain contributions	Boundary-crossing across problem spaces	Socially contextual	1.83	1.51	−17.5%	−0.042
First-contact engagements	New-tie formation	Socially contextual	2.87	2.34	−18.5%	−0.047

*Note.* Public exchange function labels follow the mapping established in Section 2.4. “Direct private support” indicates functions for which generative AI provides a relatively direct private-support pathway; “socially contextual” indicates functions that depend more heavily on contextual judgment, novel perspective, relationship formation, or serendipitous exposure. Among rows with $\beta_{2}$ estimates, the mean absolute coefficient is 0.091 for direct/partial private-support interaction types and 0.045 for socially contextual interaction types. A separate external-platform diagnostic indicated a 14.5% decline in out-of-ecosystem interactions, but that measure lacks comparable pre/post GitHub component counts and $\beta_{2}$ estimates, so it is treated as a diagnostic rather than as a decomposition row.

## Data Availability

De-identified aggregate data and replication materials are available from the corresponding author on reasonable request, where consistent with survey consent and platform terms. Raw survey responses and user-level identifiers are not publicly released.
